# Food and Force

**Published:** 1890-03-01

**Authors:** 


### THE DOCTOR'S PULPIT.

### FOOD AND FORCE.

THE value of force is as well understood in these times as the value of money. "To take a man's measure" is one of the commonest of daily experiences. Is he a clerk? How long can he work at high pressure, always turning out work of the best quality? How much guidance does he require; how much stimulus? Or, if he is left alone to do his own work in his own way, what is the general value of the completed result? Perhaps it is the literary man's force it is desired to gauge. How much originality is there in him; how much honesty; how much strength of conviction and courage in the utterance thereof? How long can he work; and with what energy? What force of character can he bring to bear upon new or difficult problems? With what degree of precision can he seize upon the main lines of an argument; and with what convincing energy can he present them to his readers?

In ages not very remote physical force would have claimed the first place in a discussion on this topic. Even now the money value of bodily health, energy, and staying power is very great. We say "money value," because everybody can appreciate that. But in almost all the important circumstances of modern life moral and intellectual energy rank higher than physical strength, and are quite beyond price. In public life, for example, to know exactly where a man stands; on what side he will generally be found; with what degree of courage, steadfastness, and onward movement he will support and maintain his convictions; this, to both friends and foes, is invaluable. Human force of any and every kind is thus seen to be a question not of second-rate, but of first-rate importance.

If money and the getting of it be one of the most urgent questions of every man's daily experience, just as much, and perhaps even more, is it true that force and the getting of it is a question of paramount consequence. Force can almost always get money; money cannot by any means always get force. If a man have but muscle and health he can delve and dig, and so put money into his pocket. Or if, having neither muscle nor health, he have the trader's skill or the writer's art, he can still command money. But if wealth, in whatever abundance, be his only possession, he may be destitute of force of every sort, even of that very elementary intellectual capacity which is required for the proper spending of his own money.

Force, then, we insist is of prime importance to man. How is it to be obtained in fullest measure? Let no one charge the writer with Materialism when he affirms that under the present conditions of human existence food is the chief agent in the production of all kinds of available force. It will readily be admitted that physical energy is mainly the product of food; but many will be prepared to deny that intellectual and moral energy are largely dependent upon food too. Yet it is not too much to say that food is almost as much the *sine quâ non* of moral and intellectual force as it is of physical. Take the example of a man totally deprived of food for eighteen or twenty-four hours; not only can he neither walk, nor run, nor ride on horseback, nor fight a battle as well as he could if he had had all his usual meals; but he cannot think, or write, or speak with the same clearness, the same energy, the same variety and wealth of illustration. Nay, more, his capacity for forming sound moral judgments will be impaired, and the tone and temper of his mind on moral questions will be lowered, at least to this extent, that he will find it much harder to be just, much harder to be patient, much harder to be kind and reasonable, than he does when his appetite and the satisfaction of it are in their normal state. That which is true of the man suddenly deprived of his food for a certain number of hours is equally true of the man or woman who from any cause whatever gradually loses the power of taking and assimilating that quantity and kind of food which he required in normal health. The physical force, it is evident to all, is diminished, and that in the exact proportion to the quantity of food he fails to assimilate. But does the same hold good of intellectual energy? Undoubtedly! The man who takes less food than his system in a normal state of health requires cannot use his brain either so energetically, or so skilfully, or for so long a time as he could if he ate and properly digested that which normal health would demand.

Many who will go thus far with us will hesitate at the proposition that moral force, like physical and intellectual, is also dependent upon food. But in considering such a question we must be guided by facts and not by prepossessions and prejudices. Not only is the power of doing all kinds of agreeable and disagreeable duties, and of carrying out all kinds of energetic philanthropies, impaired by the daily habit of taking and digesting too little food, but the desire and the will, the very centre and source of moral effort, are diminished in energy just as much as the power. Do we then affirm that a man or a woman must necessarily be made immoral by a long course of dyspepsia and insufficient food? By no means! But we do affirm that as a practical moral force and as a practical intellectual energy, the man who habitually takes less food than he would take if his health were normal is enfeebled and less efficient in exact proportion to the diminution in his normal quantity of food.

There are certain estates in Kent known to the writer which are becoming more exhausted and more valueless every day. By consequence the landlords are becoming poorer and poorer. If the process continues, the old families represented by such landlords will inevitably be dispossessed, and instead of holding their own among the more or less distinguished few, they will be merged in the general population, and will probably sink to a very low level. Everybody can see that such people are either exceedingly vicious or exceedingly incompetent. It is generally acknowledged that to waste one's patrimony, or so to mismanage it as to make it waste itself, is a disgraceful thing. But what does the patrimony of the great mass of mankind consist of? Usually of nothing else but force, and the opportunity of producing it: force intellectual: force physical: force moral: or of all three combined. He who impairs these wastes his patrimony. Many a man lavishes away his intellectual energy as extravagantly as the most foolish gambler squanders the gold that somebody else has accumulated. Many a clergyman spends his moral force so freely that there comes a time when his faith has hardly energy enough to preserve its hold on a personal deity. These persons are mentally as incompetent as the landed proprietor who muddles away his estate. They spend their force, but they do not replenish the exchequer. They have not science enough to understand, or faith enough to believe on the strength of another man's word, that food eaten and digested is the source and measure of every man's efficient energy who has to do his work by means of a body and a brain. Under what conditions pure intelligence may do its work it is impossible for us to say. Man in this world is not a pure intelligence, but a moral agent associated with a material organism, and capable of working through it, and through it only. That material organism lives by food, its energy is measured by the quantity of energy stored up in the food it takes. It cannot live in any other way; it cannot spend any more energy than the food it assimilates is capable of yielding. These are scientific certainties which nobody can deny; and the conclusion of the whole matter is that in the long run appetite and digestion will prove themselves masters of every field.

